# Contributions of charge-density research to medicinal chemistry

**DOI:** 10.1107/S2052252514018867

**Published:** 2014-09-23

**Authors:** Birger Dittrich, Chérif F. Matta

**Affiliations:** a Institut für Anorganische und Angewandte Chemie, Martin-Luther-King-Platz 6, 20146 Hamburg, Germany; b Department of Chemistry and Physics, Mount Saint Vincent University, Halifax, Nova Scotia B3M 2J6, Canada; c Department of Chemistry, Dalhousie University, Halifax, Nova Scotia B3H 4J3M, Canada; d Department of Chemistry, Saint Mary’s University, Halifax, Nova Scotia B3H 3C3, Canada

**Keywords:** charge-density research, medicinal chemistry, drug design, invariom, Hansen–Coppens multipole model, quantum theory of atoms in molecules

## Abstract

This article reviews efforts in accurate experimental charge-density studies with relevance to medicinal chemistry. Initially, classical charge-density studies that measure electron density distribution *via* least-squares refinement of aspherical-atom population parameters are summarized. Next, interaction density is discussed as an idealized situation resembling drug–receptor interactions. Scattering-factor databases play an increasing role in charge-density research, and they can be applied both to small-molecule and macromolecular structures in refinement and analysis; software development facilitates their use. Therefore combining both of these complementary branches of X-ray crystallography is recommended, and examples are given where such a combination already proved useful. On the side of the experiment, new pixel detectors are allowing rapid measurements, thereby enabling both high-throughput small-molecule studies and macromolecular structure determination to higher resolutions. Currently, the most ambitious studies compute intermolecular interaction energies of drug–receptor complexes, and it is recommended that future studies benefit from recent method developments. Selected new developments in theoretical charge-density studies are discussed with emphasis on its symbiotic relation to crystallography.

## Abstract 2

Contributions of experimental and selected theoretical charge-density research to medicinal chemistry are reviewed; combining experimental methods from high-resolution small-molecule and macromolecular crystallography with theory proves to be fruitful.

## 1. Abbreviations   

ADPs: anisotropic displacement parameters

CD: charge density

DI: delocalization index

EDD: electron density distribution

EDWCM: electron-density-weighted connectivity matrix

ELMAM2: experimental library multipolar-atom model

EP/MM: exact potential and multipole methods

ESP: electrostatic potential

GID: generalized invariom database

IAM: independent-atom model

KEM: kernel energy method

LDM: localization–delocalization matrix

LI: localization index

QTAIM: quantum theory of atoms in molecules

SBFA: supramolecular synthon-based fragments approach

TLS: translation, libration and screw motion

UBDB: University at Buffalo Databank

XRD: X-ray diffraction

## 2. Introduction   

Research challenges in biomedical and medical research are fascinating and intrigue many researchers. In how far small-molecule (Wouters & Ooms, 2001 ▶) and macromolecular (Anderson, 2003 ▶) XRD can contribute to processes such as drug design and development is the subject of intensive research (Davis *et al.*, 2003 ▶). Contributions to and success stories of structure-based drug design were also discussed in a textbook (Klebe, 2009 ▶). The focus of this article is experimental CD research, historically the specialized area of small-molecule X-ray crystallography that focuses on high accuracy, and its relevance to medicinal chemistry. Since a comprehensive review article covering biophysical and biological properties derived from *theoretical* CD has been published very recently (Matta, 2014 ▶), only experimental CD and some selected aspects of theoretical CD research will be covered here. For an introduction to general CD research, the monographs by Tsirelson & Ozerov (1996 ▶) and Coppens (1997 ▶), and the review articles by Spackman & Brown (1994 ▶), Spackman (1998 ▶), Koritsánszky & Coppens (2001 ▶) and Stalke (2011 ▶) are recommended. The essence of experimental CD work is that one measures ρ(**r**), the EDD of a molecule in the solid state, more specifically a molecule surrounded by countless other molecules in a close packing arrangement — unlike in quantum chemistry, where usually only the EDD of a molecule in the gas phase is considered. Experimental CD research commenced with an *X*—N difference electron density study on *s*-triazine (Coppens, 1967 ▶). For the first time, it allowed visualization of the rearrangements of ρ(**r**) due to chemical bonding, which was predicted to be possible by Debye (1915 ▶). CD research quickly developed to be one of the research areas at the forefront of method development in XRD, pushing this branch of science forward in the 1970s to the 1990s. The coming of age of classical CD studies, where multipole population parameters of aspherical scattering factors are adjusted to high-resolution Bragg data by least-squares refinement, was declared a decade ago (Coppens, 2005 ▶) and there is, in principle, no limit on the size of the system studied.^1^ The tremendous drive and progress in protein crystallography superseded the leading role of CD in method development with completely opposite problems to be solved, *i.e.* handling low-resolution rather than ultrahigh-resolution (Jelsch *et al.*, 2000 ▶) data. Nevertheless, both areas are still advancing the capabilities of XRD in opposite but complementary directions with respect to challenges in medicinal chemistry. The coming of age of low-resolution protein crystallography has equally been declared (Brünger, 2006 ▶) and developments in structural biology have empowered structural biologists in their endeavors, which has led, for example, to the award of the 2009 Nobel Prize in Chemistry to Ada Yonath (Yonath, 2010 ▶), Thomas Steitz (Steitz, 2010 ▶) and Venkatraman Ramakrishnan (Ramakrishnan, 2010 ▶) for elucidating the structure and function of the ribosome.

## 3. Combining experimental CD and protein crystallography   

One topic of interest shared by CD studies and protein crystallography is the experiment because both require the best attainable data to give the most reliable answer to a particular structural problem. Here both research areas, CD (Coppens *et al.*, 1974 ▶; Larsen, 1995 ▶; Stalke, 1998 ▶; Hardie *et al.*, 1998 ▶) and macromolecular crystallography (Hope, 1990 ▶; Garman, 1999 ▶; Petrova *et al.*, 2006 ▶; Chinte *et al.*, 2007 ▶) usually rely on the use of low-temperature data collection and synchrotron radiation (Coppens, 1992 ▶; Helliwell, 1998 ▶); for example, when trying to identify the protonation state of a residue (Dauter *et al.*, 1997 ▶), when collecting multiple anomalous dispersion data for solving the phase problem (Dauter *et al.*, 1998 ▶), or when data devoid of strong bias from extinction and absorption of metal containing coordination complexes are collected to the highest possible resolution with hard X-rays (Schmökel *et al.*, 2013 ▶).

Distinction between small-molecule and macromolecular crystallography can appear such as a divide not unlike that between neighboring branches of science, *e.g.* biology and chemistry.^2^ This divide is counterproductive for advancing the possible impact of CD research on medicinal chemistry, or to tackle challenges in macromolecular crystallography that are usually not encountered by small-molecule crystallographers; both rest on the same experimental foundations, mathematical background and underlying physical effects. On the contrary, combining the expertise of both branches of crystallography is fruitful to study research questions of medical relevance. One example is the use of on-the-fly computation of three-dimensional Fourier difference electron density maps in macromolecular crystallography (Emsley & Cowtan, 2004 ▶), and their subsequent implementation in small-molecule software (Hübschle & Dittrich, 2011 ▶; Hübschle *et al.*, 2011 ▶), another avoiding different use of symbols and definitions for atomic displacement parameters (Trueblood *et al.*, 1996 ▶).

More recently, both small-molecule and macromolecular crystallography are being applied together in research relevant to medicinal chemistry (Dominiak *et al.*, 2009 ▶; Malińska *et al.*, 2014 ▶), and it is these developments and challenges of such applications that are both the focus and culmination of this article. Such studies should ultimately rely on accurate structural knowledge of *both* receptor and drug molecules, and therefore require the techniques and methodologies in both research areas to be combined.

## 4. Developments of CD work – relevant to medicinal chemistry?   

The focus on high accuracy, by improving experimental conditions, equipment and choice of specimen, can be seen as both a blessing and a curse to experimental CD work. On one hand, it is necessary to aim for the best possible experiment (Seiler, 1992 ▶; Destro *et al.*, 2004 ▶; Zhurov *et al.*, 2008 ▶) using the most sophisticated model to study a particular research question. On the other hand, a methodology that *requires* the best possible experimental result excludes studying many interesting research questions of broader relevance where experimental requirements (Blessing & Lecomte, 1991 ▶; Koritsánszky *et al.*, 1998 ▶) cannot be met. This was the starting point for the development of scattering-factor databases (Brock *et al.*, 1991 ▶), initially to improve the accuracy of ADPs (Jelsch *et al.*, 1998 ▶; Dittrich *et al.*, 2008 ▶), then to improve structural least-squares refinement of oligopeptides (Pichon-Pesme *et al.*, 1995 ▶) and small proteins (Jelsch *et al.*, 2000 ▶), and ultimately light-atom structures in general (Dittrich *et al.*, 2004 ▶, 2013 ▶). A second, equally valid starting point was to obtain properties^3^ and then intramolecular interaction energies from aspherical atoms (Li *et al.*, 2002 ▶; Volkov, Li *et al.*, 2004 ▶; Jarzembska & Dominiak, 2012 ▶). Most recently such an analysis was applied to macromolecular systems. We here consider these methods, mostly developed in the last two decades, to be part of the field of CD research, as the unifying aim of high accuracy is shared, although their experimental requirements are different to classical CD work: scattering-factor libraries^4^ providing the EDD can be applied to data sets of normal resolution in the refinement of positions and atomic displacement parameters (speaking from the viewpoint of a small-molecule crystallographer, *i.e.* data sets that fulfill the requirements of the journal *Acta Cryst. Section C*: 25° in 2θ with Mo *K*α radiation, which is approximately 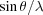 = 0.6 Å^−1^ or *d* = 0.84 Å), as the multipole populations of the scattering factors do not need to be refined anymore but are used unchanged as tabulated. Hence, using a fixed scattering factor of the pseudoatom model (Stewart, 1976 ▶) as modified by Hansen & Coppens (1978 ▶) opens up the field to such research problems, where the data resolution and quality required are simply not currently measurable. Such approaches therefore substantially increase the reach of CD research. Property calculations using the EDD calculated from the tabulated multipole parameters to obtain properties require only a set of molecular coordinates, in principle also from data of lower resolution or methods other than XRD. Whether scattering-factor databases are also suitable for refinement in protein crystallography, where atomic resolution is already considered high, and whether coordinates from low-resolution refinement are good enough for obtaining reasonable properties will be important questions that are discussed below. We will first look at classical CD studies with relevance to medicinal chemistry before we move on to such applications of databases.

## 5. Classical CD research in medicinal chemistry   

Classical CD studies of drug *and* macromolecular receptor are impossible as long as truly ultrahigh-resolution data become available for macromolecules and until the challenge of treating disorder has been successfully tackled. Because such ideal situations are unavailable, it is constructive to first study separately a single, active small-molecule pharmaceutical ingredient. Many such studies have been performed (Howard *et al.*, 1995 ▶; Flaig *et al.*, 2001 ▶; Hibbs *et al.*, 2003 ▶; Ghermani *et al.*, 2004 ▶; Destro *et al.*, 2005 ▶; Soave *et al.*, 2007 ▶; Rajalakshmi *et al.*, 2014 ▶), more recently also on a pair of polymorphs (Overgaard & Hibbs, 2004 ▶; Nelyubina *et al.*, 2010 ▶), a series of pharmaceutically active molecules (Zhurova *et al.*, 2006 ▶; Parrish *et al.*, 2006 ▶; Yearley *et al.*, 2007 ▶; Zhurova *et al.*, 2009 ▶; Grabowsky *et al.*, 2008 ▶) and anion–receptor complexes (Kirby *et al.*, 2014 ▶) in comparative CD studies, to give just a few examples. For further illustration a study on two penicillin molecules, one active and one inactive (Wagner *et al.*, 2004 ▶), will now be discussed in slightly more detail. A question underlying this and several other studies was whether the experimental EDD would provide an indication on activity from bond topology following Bader’s QTAIM (Bader, 1990 ▶). This turned out not to be the case because the experimental topology was very similar within the standard deviation (Dittrich *et al.*, 2002 ▶) for similar bonds in an active and a non-active penicillin derivative; similarity was defined as sharing the same chemical environment. The ESP (Náray-Szabo & Ferenczy, 1995 ▶) proved rather more useful (Stewart, 1979 ▶; Stewart & Craven, 1993 ▶). Encouragingly an agreement between theoretical and experimental ESP, with the latter being slightly more extended, could be established (Dittrich *et al.*, 2000 ▶), and for the active penicillin the ESP confirmed the established mechanism of action. Many subsequent studies of the ESP applied an analysis introduced by Politzer *et al.* (2001 ▶) in order to be able to identify and quantify characteristics of particular classes of compounds. Still, classical CD studies can be time-consuming, require a non-disordered structure and high crystal quality. Often only a few out of a series of compounds fulfill these specific requirements. A subsequent contribution to this research area relied on the invariom database, benefitting from already measured conventional data. This allowed 12 X-ray data sets of nine active fluoroquinolones to be studied in a reasonably short time using published, remeasured and newly determined structures with different crystal quality (Holstein *et al.*, 2012 ▶). Although not strictly an experimental CD study anymore because the EDD is taken from the database, having a larger sample of molecules at hand that were treated in a consistent manner provided more insight: active molecules sharing the same mechanism of action did also show a very similar ESP for the same protonation state. The ESP derived from several scattering-factor databases has recently been compared and was found to agree well between all of them (Bąk *et al.*, 2011 ▶). It can be considered that such methodology is established and reliable for studies on small molecules. However, to understand biological processes in full detail both the accurate EDD of small molecule drug and macromolecular receptors are required, and dynamic processes also need to be taken into consideration.

An important aspect with respect to ESPs and other properties derived from classical CD studies is undetected disorder because multipole parameters correlate with the site occupancy of an atom in question, thereby invalidating the EDD of a disordered atom and influencing its environment. It has been shown that properties such as the ESP derived from an EDD where rotational or other subtle disorder has not been spotted become unreliable or in error (Dittrich, Warren *et al.*, 2009 ▶; Bąk *et al.*, 2009 ▶). Here the solution to arrive at the correct result is reverting to theory and to rely on database parameters for populations of those atoms affected (Holstein *et al.*, 2010 ▶).^5^ The example of 2-methyl-4-nitro-1-phenyl-1*H*-imidazole-5-carbonitrile shows how easy it is to overlook, for example, rotational disorder of a methyl group (Poulain-Paul *et al.*, 2012 ▶).

**Figure 1:**
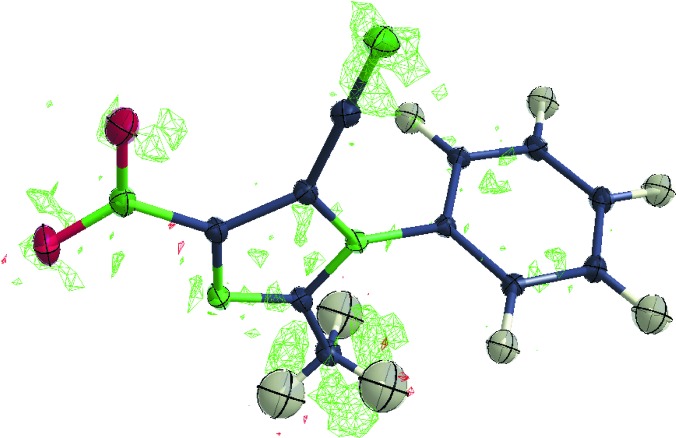
If undetected, rotational disorder can lead to erroneous properties derived from experimental CD studies. Useful for detecting it are experiment-minus-invariom difference densities such as the one shown above for 2-methyl-4-nitro-1-phenyl-1*H*-imidazole-5-carbonitrile. Green (positive) and red (negative) iso-surface meshes of the Fourier difference EDD at 0.1 e Å^−3^.

An analysis with an experimental-minus-invariom difference density (Dittrich *et al.*, 2007 ▶) using the authors’ deposited data clearly shows disorder to be present (Fig. 1 ▶). This matters because rotational disorder can also bias the experimental dipole moment, and this might explain some of the differences observed between experiment and theory.

In summary, the investigations cited in this section and many other studies of this kind show that experimental CD work does provide valuable information on molecules in the crystal and their properties, but the effort involved is often considerable. The outlook is positive though, and we can expect the time of both modeling process and experiment to be further reduced in the future (Hübschle *et al.*, 2007 ▶; Schürmann *et al.*, 2012 ▶) as previously anticipated (Luger, 2007 ▶).

## 6. Interaction density – relevant fundamental research   

Interaction density^6^ is relevant to medicinal chemistry because crystallization is a molecular recognition process. Crystallization (and interaction density) can be seen as an idealized situation that is analogous to drug–receptor interactions and the redistribution of electron density of a drug molecule in the active site. Hence, it would be of considerable interest to understand whether or not the process of a molecule becoming polarized helps crystallization, or is just a consequence of it. What one could learn from answering this question would be directly relevant to other molecular recognition processes, here, for example, drug–receptor interactions.

The starting point in studying interaction density from a CD point of view was Bader’s QTAIM and difference electron density studies to understand and visualize the redistribution of ρ(**r**) of a hypothetical molecule in the gas phase and one that is part of the crystal environment (Gatti *et al.*, 1994 ▶; Spackman *et al.*, 1999 ▶). Unfortunately, further studies (Dittrich & Spackman, 2007 ▶) were complicated by technical problems, for example, the limited flexibility of the single-zeta Hansen–Coppens multipole model (Volkov & Coppens, 2001 ▶) using parameters up to hexadecapoles. Here a basis-set description, as available in the program *Tonto* (Jayatilaka & Grimwood, 2003 ▶), has advantages in reproducing fine features of the EDD (Dittrich *et al.*, 2012 ▶). However, the measurement of a lot more reflections than is currently possible would be required for an experimental CD study employing even more parameters. Although Hansen and Coppens have optimized their model to describe covalent bonding of light-atom structures, further advances in this area will continue to require a considerable amount of work to explain differences that become increasingly small.

The conclusion of studies of interaction density was that the crystal field can indeed cause detectable redistributions of the EDD in a molecule that is part of a crystal when compared with its gas-phase counterpart with identical structure and conformation. However, these differences are certainly small (in the range of 0.25 e Å^−3^), and just at the level where even good data sets become noisy and the phases of noncentrosymmetric structure (Spackman & Byrom, 1997 ▶) become affected by experimental errors (Souhassou *et al.*, 1991 ▶). While we still think that a qualitative experimental measurement is indeed feasible with low-temperature data because studies with model data (not taking into account thermal motion) predicted that measurements should be possible, other authors have been more skeptical (de Vries *et al.*, 2000 ▶). In a next step, energetic contributions of the electron density redistribution could be estimated, something we think is a worthwhile endeavor. For now, it remains unclear how important interaction density is for crystallization, and likewise how important electron density redistribution is for drug–receptor interactions.

## 7. Current challenges in high-resolution macromolecular crystallography   

There are many interesting directions for method development in macromolecular crystallography and these have already been discussed elsewhere (Adams *et al.*, 2013 ▶). One of the most pertinent problems in macromolecular crystallography with respect to CD work is the challenge of accuracy, which is directly related to data resolution. Whereas almost all protein structures are determined at resolutions above 0.5 Å, classical CD methodology cannot be applied for studying such structures. Our experience shows that even macromolecular data sets that formally fulfill the requirements of CD research (Blessing & Lecomte, 1991 ▶), or those getting close to fulfilling these requirements, are usually unsuited owing to the numerous disordered atoms, and there is currently only one such example of the small protein crambin (Schmidt *et al.*, 2011 ▶) where a resolution below 0.5 Å has been reached.^7^ Despite several review articles that painted the future to be bright (Schmidt & Lamzin, 2002 ▶; Vrielink & Sampson, 2003 ▶; Petrova & Podjarny, 2004 ▶), achieving resolutions in macromolecular crystallography such as those that are routinely reached in small-molecule crystallography more frequently would be highly desirable.

Another way to improve the accuracy of macromolecular structures, while not relying on experimental improvements, is to use a more sophisticated scattering factor model beyond the IAM usually used for refinement and subsequent analysis. When using fixed aspherical scattering factors resolution requirements as they apply in CD research can be reduced to a certain degree (*d* = 0.84 Å) (Dittrich, Hübschle *et al.*, 2009 ▶), but a better model only makes physical sense for macromolecules when features of valence EDD can be observed (Afonine *et al.*, 2004 ▶). Although there are no additional parameters being added with fixed aspherical scattering factors (unlike in classical CD refinement), this approach should at least not make the fit to the experimental Bragg intensities worse even when this requirement is not entirely met (Housset *et al.*, 2000 ▶), and it has been shown to lead to significant improvements in some systems (Guillot *et al.*, 2008 ▶; Dittrich *et al.*, 2010 ▶; Pröpper *et al.*, 2013 ▶) similar to what is seen in small-molecule structures. There is currently only one program designed (and suitable) for such refinements, because it is, for example, capable of using restraints and has also implemented the Hansen–Coppens multipole model, and that is the program *Mopro* (Guillot *et al.*, 2001 ▶; Jelsch *et al.*, 2005 ▶).

An alternative model to improve macromolecular structures at ultrahigh resolution is based on the IAM, but additional parameters are required in the form of spherical scatterers for bonding and lone-pair electron density (Hellner, 1977 ▶). This approach already had some utility (Afonine *et al.*, 2007 ▶) and one strong point is the ease of program implementation; an interesting recent study added atomic charges to interatomic scatterers (Nassour *et al.*, 2014 ▶). An alternative way to improve macromolecular structures is the combination of force-field calculations with structure refinement as in the program *CNS* (Brünger *et al.*, 1998 ▶), and to improve the force-field description further by including polarization. This has recently been shown to be possible (Schnieders *et al.*, 2009 ▶). Ultimately, the contribution of the experiment remains the most important one, and the above-mentioned methods and programming improvements only work and show their utility most convincingly with the best current macromolecular data sets.

Currently, the only possibility to solve the challenge of positional inaccuracy and disorder with lower resolution data is to include chemical knowledge in the form of restraints, or to use constraints, for example, in the form of fixed aspherical scattering factors. Restraints (Engh & Huber, 1991 ▶) have been used in macromolecular crystallography for decades, since classical least-squares refinement does not always provide a physically correct answer, most obviously when a side chain is dynamically disordered or when there are too many or highly correlated least-squares parameters. Although the use of restraints is well established in low-resolution protein refinement, it is counterintuitive for small-molecule crystallographers used to high resolution, and is conceptually hard to swallow, especially for those who follow the ideal of measuring experimental CD, where accurate measurements are the center of interest.

It could be misconstrued that for truly high resolution, protein data restraints are not required because the large number of reflections leads to favorable overdetermination for least-squares refinement. This is a misconception because macromolecules almost always contain a substantial part of dynamically disordered solvent and side chains. When such disorder is present restraints are needed for protein data at truly high resolution. On the other hand, the well behaving parts of such a macromolecule would not need restraints at all and permit free parameter adjustment. However, refined distances might then disagree with the conventional Engh–Huber restraints that were derived from the IAM that has been shown to lead to inaccurate positional parameters (Coppens *et al.*, 1969 ▶). Therefore, these widely used restraints are outdated when used in combination with aspherical scattering factors. It would hence be highly desirable to have a new set of bond-distance, bond-angle and other restraints (Thorn *et al.*, 2012 ▶) available (Jaskolski *et al.*, 2007 ▶) that agree both with neutron diffraction refinements (Gruene *et al.*, 2014 ▶), with theoretical computations and therefore also with refinement results from incorporating aspherical scattering factors. Structures refined with newly developed restraints for use with aspherical scattering factors should then also give more accurate results in subsequent analysis of their intermolecular interaction energies (see below). Last but not least, another important point is that new restraints are required not only for the constituting chemical environments of the protein or DNA macromolecules, but also for solvent molecules, all possible ligands or other (small molecule) cofactors (Kleywegt, 2007 ▶).

Another challenge is the treatment of hydrogen atoms, which has been the subject of continuous studies over the decades (Stewart *et al.*, 1965 ▶). Hydrogen atoms have a weak scattering contribution, mainly in the low-order region of the diffraction pattern, and the accurate determination of their positional and displacement parameters is therefore a fundamental problem of single-crystal XRD (Engler *et al.*, 2003 ▶; Munshi *et al.*, 2008 ▶; Jayatilaka & Dittrich, 2008 ▶; Hoser *et al.*, 2009 ▶). Historically, neutron diffraction data have often been collected in parallel to collecting small-molecule high-resolution XRD data. However, such studies have been infrequent because requirements on crystal volume have historically been difficult to fulfill. Despite improvements in technology, their number has not increased because the number of machines available for such experiments continues to be limited. Hence until today, when analogous studies are being applied in macromolecular crystallography (Afonine *et al.*, 2010 ▶), such experiments are not being carried out as often as they should be.

The most noteworthy development in small-molecule crystallography, in this context, is the combination of X-ray and neutron scattering experiments and analysis to give spin-resolved electron distributions (Deutsch *et al.*, 2014 ▶). Nevertheless, improvements in the hydrogen-atom treatment remain possible without invoking neutron diffraction and it has recently been shown that even the standard riding hydrogen treatment can be improved (Lübben *et al.*, 2014 ▶); we plan to continue this work by estimation of ADPs, following up on earlier work by Madsen (2006 ▶) and Whitten & Spackman (2006 ▶). Frequency computations of the model compounds in the invariom database will be combined with a TLS fit for that purpose, thereby avoiding additional least-squares parameters for refinement of hydrogen atoms.

A recurring theme in this article is accuracy; further improving the experimental data quality of macromolecules will continue to be helpful and important. Modern detectors (Broennimann *et al.*, 2006 ▶; Toyokawa *et al.*, 2010 ▶) certainly help in achieving this goal. However, the study of small molecules, where crystals remain stable and do not show much radiation damage during the course of measurement, can certainly help us to understand how to improve data quality with new equipment. Low-order data are especially relevant for macromolecular structure determination in the presence of a disordered solvent and for observing deformation EDD, and we frequently observe problems with pixel detectors. The article by Dauter (2003 ▶) showed how high-quality low-order data for macromolecular structures can be collected with CCD detectors. Such a study remains to be repeated for the new generation of pixel detectors that are replacing CCD detectors at synchrotrons.

## 8. Opportunities and challenges of combined CD and macromolecular work in medicinal chemistry   

A long term aim in CD research has been to extract physical properties, such as, for example, the interaction energy, from the experimental EDD in the solid state. This research has had a long history and a readable introduction to it has recently been provided as a book chapter (Dominiak *et al.*, 2012 ▶) where the different approaches are comprehensively covered and compared, while also giving account of existing literature. The computation of the interaction energy (or the electrostatic contribution to the interaction energy) from experimental or database electron density may be the most ambitious but also the most promising goal in combining CD and macromolecular crystallography. Analogous to the interaction density that is smaller than the bonding and core electron density, the interaction energy is orders of magnitude smaller than the energy of the molecules themselves. Another similarity is that the interaction energy is a feature of the crystal packing, and is an energy difference. Many studies have been carried out to obtain interaction energies in small-molecule systems (Spackman & Weber, 1988 ▶; Abramov *et al.*, 2000*a* ▶,*b* ▶; Li *et al.*, 2002 ▶; Soave *et al.*, 2007 ▶; Bouhmaida *et al.*, 2009 ▶). These pivotal studies provided very useful experiences, including an assessment of accuracy and the EP/MM approach (Volkov, Koritsánszky & Coppens, 2004 ▶) currently seems closest to a user-friendly implementation of the concepts involved. Being able to carry out high-throughput studies of a series of related drug compounds with different affinity for quantitative property screening is certainly a future requirement on the side of small-molecule crystallography. Another interesting new development is to also assess weak intermolecular interactions from database electron density (Nelyubina *et al.*, 2014 ▶).

Much progress has also been made concerning interaction energies of *macromolecular* drug–receptor complexes from databases. Parameters describing the electron density of both the building blocks for proteins (Domagała *et al.*, 2012 ▶) and DNA (Jarzembska & Dominiak, 2012 ▶) and many other possible chemical environments (Dittrich *et al.*, 2013 ▶) are available in the above-mentioned scattering factor databases, and the stage is set for studying drug–receptor interactions quantitatively. The most eye-catching current studies in the area have been on neuraminidase (Dominiak *et al.*, 2009 ▶) and sunitinib in complexes with different kinase receptors (Malińska *et al.*, 2014 ▶) using the UBDB (Jarzembska & Dominiak, 2012 ▶), which was designed to reproduce as well as possible the theoretical interaction energies with the Hansen–Coppens multipole model, whereas the aim in developing the invariom database was to provide better structures. Both aims are closely related, because only with good positional parameters and deconvoluted ADPs can accurate properties be obtained. Hence, protein structures of limited resolution can be suspected not to be a very good starting point for providing accurate interaction energies. This is why we advocate for continuous improvements in high-resolution protein refinement, for example, by testing re-refinement with deposited data, or better, with newly measured X-ray data to the best possible resolution. The work on aldose reductase (Guillot *et al.*, 2008 ▶) is pioneering especially in this respect, because for the first time a real enzyme with hundreds of amino acids was studied at a very respectable resolution, even together with an inhibitor, and aspherical-atom refinements were carried out on the system. Suspected (Lichtenthaler, 1994 ▶) electrostatic complementarity (Muzet *et al.*, 2003 ▶) has been confirmed and will remain to be a very useful concept. We share the aim of using aspherical scattering factors in refinement, and have carried out two related studies on the two peptide antibiotics trichotoxin A50E (Dittrich *et al.*, 2010 ▶) and thiostrepton (Pröpper *et al.*, 2013 ▶) with the *XD* suite of programs (Volkov *et al.*, 2006 ▶), systems that are however an order of magnitude smaller than aldose reductase. Another aspect regarding the calculation of interaction energies is that the Hansen–Coppens density model may, without modification, not be sophisticated enough to produce energies with a good degree of accuracy (Bąk *et al.*, 2011 ▶). Nevertheless, despite the challenges encountered, work on aldose reductase, trichotoxin A50E, neuraminidase, thiostrepton and sunitinib are currently the best efforts that can be made and guide the way.

## 9. Complementarity and synergy of experiment and theory   

Nowadays, it is common practice to support experimental determinations of geometries and electron densities with theoretical calculations both in the isolated molecular vacuum phase and in the crystalline phase. This is so because the object of the study, ρ(**r**), is accessible from both ends: theory – through the calculation of the many-electron wavefunction 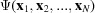, where 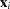 are the space and spin coordinates of the *i*th electron, and experiment – as discussed above from the resolution of the crystallographic phase problem and subsequent modeling of the resulting electron density.

The EDD, the ESP, the geometries, and all ground- and excited-state properties are all uniquely mapped to one another as has recently been emphasized (Matta, 2014 ▶) owing to the operation of the Hohenberg–Kohn theorem (Hohenberg & Kohn, 1964 ▶). Structures, EDD and ESP are all three accessible from both theory and state-of-the-art experiment and with comparable precisions leading to a very desirable synergy of theory and experiment. Theory can supply additional molecular descriptors obtained from the full density matrix (normally accessible only from calculations) rather than just the diagonal elements of this matrix that can be fitted to experimental scattering data. In the remainder of this short review, we select a few examples of the synergy and complementarity of quantum chemical theory and X-ray crystallographic experiment for the sake of illustration rather than to provide an exhaustive review.

*Ab initio* methods scale rapidly with the size of the system, and as a result they often cannot be applied to very large biological molecules of importance to medicinal chemistry. A fragmentation solution to this problem, based on Bader’s QTAIM, has been proposed and demonstrated to reproduce the *ab initio* results at a fraction of the computational costs using a series of morphine analogs (opioids) (Matta, 2001 ▶). This method is termed the ‘buffered fragments approach’ because the properties of the large system are obtained from calculations on small fragments embedded in an appropriate (buffer) electronic environment similar to the environment in the target molecule. The fragments are extracted from their environments at their zero-flux surfaces (Bader, 2001 ▶) and then combined to reconstruct the properties of the target molecule. Such atomic partitioning of the electron density has also been applied to EDD from single-crystal XRD and the submolecular transferability that was previously demonstrated on the basis of theoretical calculations (Matta, 2001 ▶) has likewise been demonstrated for molecules of the same opioid family based on experimentally determined EDD. Scheins *et al.* (2005 ▶) have shown how to reconstruct an approximation to the experimentally derived EDD of morphine from buffered experimental fragments (Scheins *et al.*, 2005 ▶) in concordance with the theoretical counterparts. The goal of Luger *et al.* extends beyond the particular chosen (opioids) systems to a much broader proof of principle that the buffer fragments methods can be used to obtain the EDDs of large molecules of biological significance, an important contribution in efforts to circumvent the experimental inaccessibility of the electron densities of numerous large molecules of extreme biological and pharmacological importance. It is noted in passing that the buffered fragments reconstruction applies to both scalar and vector properties. Examples of the former include molecular volumes, and atomic and group charges (Matta, 2001 ▶; Scheins *et al.*, 2005 ▶), and examples of the latter include the dipole moment or dielectric polarization (Bader, 2002 ▶; Bader & Matta, 2001 ▶).

**Figure 2:**
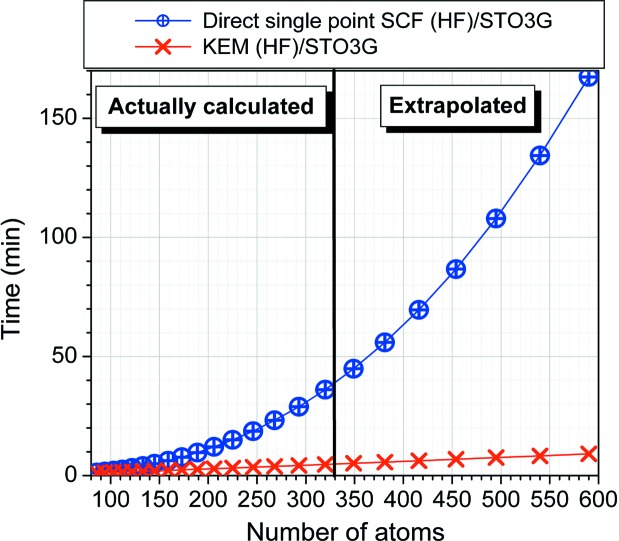
Comparison of total CPU times for converging single-point HF/STO-3G full molecule and KEM calculations on the same processor. Reproduced from the paper by Huang *et al.* (2014 ▶) with permission of the copyright owner Elsevier 2014.

Another approach, developed by Huang, Massa and Karle, is termed the kernel energy method (KEM) and it represents another area of synergy between crystallography and *ab initio* quantum mechanics. In this approach, X-ray crystallography supplies the experimental coordinates and theory supplies the fast and accurate estimate of the total energies, interaction energies, stacking energies and binding energies. This energetic dimension that is obtained from the quantum calculations using the experimental geometries is invaluable in the modeling of large biomolecules, especially with regards to host–guest, enzyme–substrate or enzyme–inhibitor interactions (Huang *et al.*, 2005*a* ▶,*b* ▶,*c* ▶, 2006 ▶, 2009 ▶, 2010 ▶, 2011 ▶, 2014 ▶; Massa *et al.*, 1995 ▶). In this method, the energy of the full system is obtained at chemical accuracy through a fragmentation scheme which is different from that described above in the buffered fragments method. The reason for the difference is that when *energies at an experimentally determined frozen geometry* are the prime sought for quantity, QTAIM cannot be used because QTAIM energies in this case will include origin-dependent contributions from the virials of the net non-vanishing forces on the nuclei. The KEM provides a fast and *extremely* accurate alternative that has been extensively tested in the past decade (Huang *et al.*, 2010 ▶). KEM partitions the large molecule into double kernels, *i.e.* fragments capped with hydrogen atoms, and which account for two-body interactions between different regions of the molecule, corrected by the subtraction of the contributions of single kernels to remove the over-counting of energies from the summations of the contributions from double kernels. The KEM energy is defined as 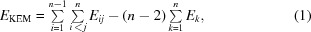
where 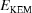 is the KEM energy of the full system, 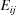 is the energy of the *ij*th double kernel, 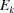 is the energy of the *k*th single kernel and *n* is the number of single kernels. Fig. 2 ▶ shows a comparison of the scaling of the CPU time for the direct calculations and the corresponding scaling from KEM calculations on the same species for a series of polypeptides. The plots in Fig. 2 ▶ show the considerable computational advantage of KEM over direct calculations particularly that differences between KEM and exact energies are typically below 1 kcal mol^−1^ for molecules with several thousands of atoms (Huang *et al.*, 2011 ▶).

Recently, an important generalization KEM (Huang *et al.*, 2005*a* ▶,*b* ▶,*c* ▶, 2006 ▶, 2009 ▶, 2010 ▶, 2014 ▶; Massa *et al.*, 1995 ▶) has been achieved, extending its domain of applicability well beyond the calculation of energies (Huang *et al.*, 2014 ▶). Indeed, KEM has been shown to be remarkably capable of the accurate prediction of response properties induced by external fields as demonstrated by the stringent test of reproducing field-induced changes in a highly delocalized finite system such as graphene. The studied properties include the change in the energy (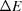) and the change in the dipole-moment components (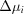, *i* = *x*, *y*, *z*) of a large, finite hydrogen-terminated graphene flake with errors practically zero for all studied response properties and all field strengths and directions (Huang *et al.*, 2014 ▶). These results enable nonperiodic quantum mechanical (cluster) calculations on extremely large systems of biological and of nanotechnological interest; impossible to achieve with existing computational technologies. Although the dipole moment is the second and a lead term in an infinite expansion, it can be anticipated that the total electron density scalar field itself can be obtained from fragments according to an equation similar in form to the constitutive KEM equation (Huang *et al.*, 2014 ▶)
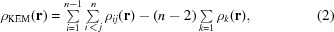
where ρ_KEM_ is the approximate KEM electron density of the full molecule to be reconstructed, ρ_*ij*_ and ρ_*k*_ are the electron densities of the *ij*th double kernel and of the *k*th single kernel, respectively, and **r** is a position vector (Huang *et al.*, 2014 ▶). Given an approximation to the density as in equation (2), all one-electron properties represented by multiplicative operators can be calculated in addition to several derived properties such as those obtained from Bader’s QTAIM.

**Figure 3:**
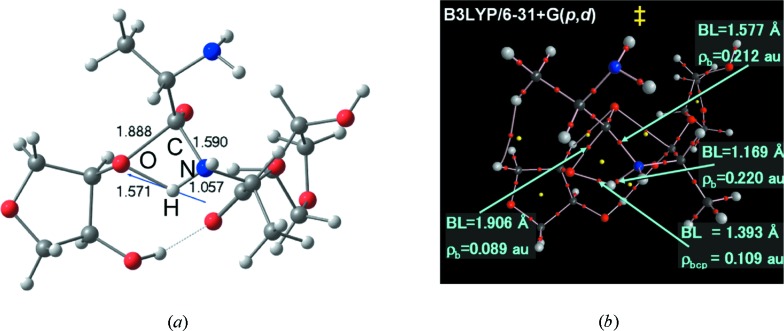
(*a*) Model of the transition state where the arrow represents the eigenvector of the imaginary frequency (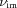 = 1084.1 *i* cm^−3^) indicating the transfer of H from the amine N to O3 [the atom labeled ‘O’ in (*a*)] of the P-site ribose sugar. The O2 hydroxyl group of the P-site tRNA (O24—H43) forms a stable hydrogen bond to the ester carbonyl group of the tRNA at the A-site (O4) (dashed line). (*b*) Molecular graph of the transition state: the large dark spheres are located at the nuclear critical points of C atoms, the large red sphere those of the O nuclei, the blue spheres are N nuclei, and the large light-gray spheres indicate the position of the H nuclear critical points. The lines of maximum electron density linking the nuclei are the bond paths and the small red dots are the BCPs. The yellow dots are the ring critical points. BL indicates bond length.

The second example of synergy between XRD experiment and theory is that which resulted in a simple mechanism for peptide bond formation in the ribosome (Gindulyte *et al.*, 2006 ▶; Massa *et al.*, 2010 ▶). Accurately determined atomic positions of the 50 most important atoms at the ribosome active site, obtained from Professor Yonath’s group, were used as the input geometry for a computational EDD investigation of the sequence of bond making and breaking in the ribosome. The sequence of steps that lead to the formation of the peptide bond in the active site of the ribosome has remained a subject of debate and disagreement. A simple mechanism has been proposed as a result of a study in which the evolution of the electron density and its topology as a function of the reaction coordinate has been elucidated in detail. The principal result is a direct mechanism instead of the oft-quoted shuttle mechanism of the peptide bond formation inside the ribosome (Gindulyte *et al.*, 2006 ▶; Massa *et al.*, 2010 ▶). In this mechanism, the amine hydrogen atom breaks away and is transferred directly to the accepting oxygen atom that simultaneously releases its attached amino acid to form the new peptide bond with the growing peptide instead of being passed to the distal oxygen which in turns passes its hydrogen atom to the active oxygen atom as in the shuttle mechanism (Fig. 3 ▶).

Very recently, a novel integration of chemical graph theory and QTAIM has been proposed (Matta, 2014 ▶; Sumar *et al.*, 2014 ▶). Matta (2014 ▶) has demonstrated that LIs and DIs obtained from QTAIM (Bader, 1990 ▶) and organized in matrix format constitute a molecular fingerprinting tool that can be used in the predictive modeling of physicochemical properties of molecules in the ground and excited states. The LI counts the number of electrons that are localized within a given atomic basin (Ω) of an atom in a molecule while the DI counts the number of electrons that are shared between two different basins (Ω_*i*_ and Ω_*j*_). The newly defined matrix, termed the localization–delocalization matrix (or the ζ-matrix, or LDM) lists the complete set of LIs [Λ(Ω_*i*_)] along the diagonal and the complete set of the DIs (divided by two), δ(Ω_*i*_, Ω_*j*_)/2, 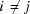, as the off-diagonal element. The LDMs are compact and efficient numerical representations of the electronic structure of molecules introduced and used as a predictive tool. It has been shown that Frobenius distances between matrix representations of the members of a series of molecules measures their molecular dissimilarities. The LDM is defined (Matta, 2014 ▶) as 
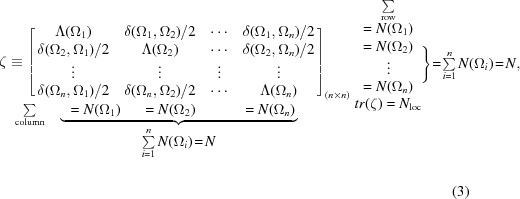
where the sum of any column or row yields the corresponding atomic population, as 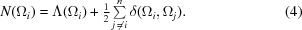
The total molecular electron population is then given by the sum of the column or row sums and can be expressed as the sum of two sub-populations
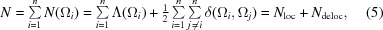
where 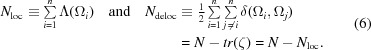



The LDM contains information on electron localization and delocalization, atomic charges [*q*(Ω) = *Z*_Ω_ − *N*(Ω), where *Z*_Ω_ is the atomic number], and interatomic distances because the DIs decay systematically with internuclear separation. The predictive value of the LDI matrices, when mathematically manipulated with the tools developed within chemical graph theory (Dimitriev, 2007 ▶; Hall & Klier, 1976 ▶; Janežič *et al.*, 2007 ▶; Todeschini & Consonni, 2009 ▶), is shown to quantify molecular similarity in hydrocarbons, and provide predictive statistical models for log *P* of the hydrocarbons (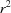 = 0.994, *n* = 4) and for the p*K*_a_ values of substituted acetic acids (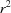 = 0.979, *n* = 7) (Matta, 2014 ▶). More recently, the LDMs have been shown to model the p*K*_a_ values of a series of *p*-substituted benzoic acids (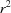 = 0.986, *n* = 14) and their UV λ_max_ values (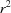 = 0.972, *n* = 8) (Sumar *et al.*, 2014 ▶). A discrepancy between the value for the p*K*_a_ = 6.03 of *p*-dimethylaminobenzoic acid (*p*-DMABA) obtained from the CRC Handbook of Chemistry and Physics (Lide, 2006 ▶, 2007 ▶) and that obtained from the ζ-matrix-based statistical model uncovered the incorrectness of the entry in the CRC Handbook, which should be corrected to 5.03 in agreement with the primary literature (Jover *et al.*, 2008 ▶), the value predicted from the ζ-matrix-based model (Sumar *et al.*, 2014 ▶).

The LDM is not directly obtainable from experiment (because the LIs and DIs necessitate the full density matrix for their calculation), nevertheless, this matrix approach is based on the partitioning of space based on the topology of the electron density into non-overlapping atoms. Furthermore, the concepts and the mathematical and numerical processing of these matrices can readily be extended to experimentally observable quantities cast in similar matrix formats. Examples of such matrices include (but are not limited to) the bond critical point (BCP) matrix termed the electron-density-weighted con­nectivity matrix (Massa, 2014 ▶), the nuclear–nuclear repulsion matrix, classical atom–atom electrostatic repulsion or attraction matrix, *etc*.

**Figure 4:**
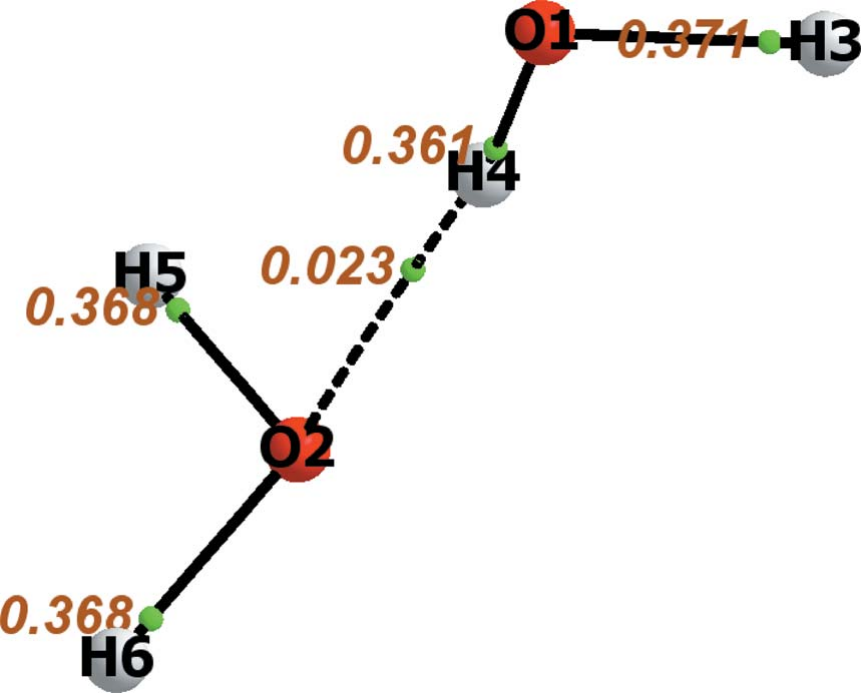
Molecular graph of the water dimer showing a set of bond paths each labeled with the value of 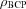 in atomic units (the small green spheres denote the locations of the BCP).

The EDWCM consists of a listing, in a matrix format, of the electron density at the BCP for every pair of bonded atoms in a molecule, and hence, the EDWCM is derivable from both experiment and theory. As in the case of the LDM, the EDWCM is symmetric, but unlike the former, all the matrix elements of pairs on non-bonded matrix elements are exactly zero since the BCP is non-existent. Fig. 4 ▶ depicts the molecular graph of a water hydrogen-bonded dimer calculated at the MP2/6-311+G(2d,2p) level of theory along with the atomic numbering scheme and the values of the electron density at the BCPs (in atomic units). Equation (7)

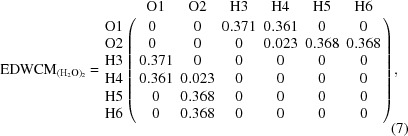
represents an EDWCM of this water dimer given the atom labeling scheme of Fig. 4 ▶ while equation (8) is the corresponding LDM
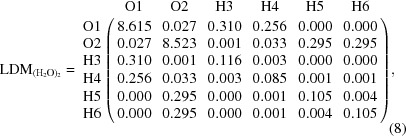
where the sums of the columns (or rows) yield: *N*(O1) = 9.208, *q*(O1) = −1.208; *N*(O2) = 9.174, *q*(O2) = −1.174; *N*(H3) = 0.430, *q*(H3) = +0.570; *N*(H4) = 0.379, *q*(H4) = +0.621; *N*(H5) = *N*(H6) = 0.405, *q*(H5) = *q*(H6) = +0.595 (where charges are expressed in atomic units). The sum of all electron populations is 20.001, the departure from the integer number of 20 electrons reflecting the overall precision of the numerical integration over the QTAIM atomic basins.

## 10. Conclusion and outlook   

It is clearly beneficial to combine methodology of both protein and small-molecule crystallography for contributing answers to research in medical chemistry. Database studies with transferable pseudoatoms can fill in many gaps inaccessible by classical CD research, and they allow computation of properties for macromolecular systems where quantum chemical *ab initio* treatment becomes unfeasible; in refinement aspherical scattering factors already help to increase the accuracy of selected macromolecular structures. As an alternative to property computation based on electron density, theoretical approaches based on the wavefunction and coordinates from XRD can provide a wealth of further properties and descriptors. Despite impressive progress a lot of work remains to be done; the key will be to combine different aspects where progress has been noticeable. To shed light on drug–receptor interactions it would be optimal to carry out a high-resolution diffraction experiment at very low temperatures (*e.g.* on a drug–receptor complex) making use of synchrotron radiation and the latest detector technology, to refine a structure with aspherical scattering factors (while evoking restraints that still need to be developed), to find a way to successfully handle disorder and to ultimately evaluate intermolecular interactions (of different conformers) in a quantitative manner. In other words, only when we are able to obtain the best possible structures can we expect that intermolecular interaction energies between macromolecules and drug molecules to be meaningful. This is also true for theoretical approaches that start from crystallographic structures to obtain energies such as KEM. If such results can be achieved, we think they will be able to provide real guidance for rational drug design.

## Footnotes

1 In practice, the increasing number of disordered atom sites with molecular size is certainly a limit.

2 One can sometimes observe misunderstandings between small-molecule and macromolecular crystallographers, maybe because either side might lack the specific experiences of the other.

3 Dipole moments and higher multipole moments (Spackman *et al.*, 2007 ▶), the molecular ESP, bond topological properties according to Bader’s quantum theory of atoms in molecules (QTAIM; Bader, 1990 ▶) and other descriptors (Gatti, 2005 ▶), the electric field gradient, electronic energy distributions (Tsirelson, 2002 ▶) and (in combination with other assumptions) intermolecular interaction energies can be obtained from experimental EDD.

4 There are currently four scattering-factor databases: the supramolecular synthon-based fragments approach (SBFA; Hathwar *et al.*, 2011 ▶), the generalized invariom database (GID; Dittrich *et al.*, 2006 ▶, 2013 ▶), the University at Buffalo Databank (UBDB; Dominiak *et al.*, 2007 ▶; Jarzembska & Dominiak, 2012 ▶) and the experimental library multipolar-atom model (ELMAM2; Zarychta *et al.*, 2007 ▶; Domagała *et al.*, 2012 ▶). While SBFA and ELMAM2 are based on refinement of multipoles from experimental high-resolution diffraction data, GID and UBDB scattering factors are derived from DFT computations; GID from optimized structures of model compounds and UBDB from single-point energy computations of selected crystal structures.

5 One has to be aware that such studies are not really experimental CD studies anymore.

6 The interaction density is the difference between the molecular EDD in the crystal and a superposition of non-interacting molecules.

7 Here the next problem to be addressed might be radiation damage (Holton, 2009 ▶).
